# Genetic Diversity of Indigenous Pigs from South China Area Revealed by SNP Array

**DOI:** 10.3390/ani9060361

**Published:** 2019-06-16

**Authors:** Shuqi Diao, Shuwen Huang, Zhiting Xu, Shaopan Ye, Xiaolong Yuan, Zanmou Chen, Hao Zhang, Zhe Zhang, Jiaqi Li

**Affiliations:** Guangdong Provincial Key Lab of Agro-Animal Genomics and Molecular Breeding/National Engineering Research Centre for Breeding Swine Industry/College of Animal Science, South China Agricultural University, Guangzhou 510642, China; saradiao@126.com (S.D.); 13422048613@163.com (S.H.); zhitingxu@126.com (Z.X.); yy623689080@126.com (S.Y.); yxl@scau.edu.cn (X.Y.); zmchen@scau.edu.cn (Z.C.); zhanghao@scau.edu.cn (H.Z.); zhezhang@scau.edu.cn (Z.Z.)

**Keywords:** Ne, genetic diversity, LD, population structure, selection signatures, South China indigenous pigs

## Abstract

**Simple Summary:**

The pig is one of the most important livestock animals, providing the majority of protein for humans. The population genetics analysis of pigs not only helps humans understand the domestication of the pig but also helps breeders in the genetic improvement of pigs. In this study, the population genetics of 11 pig breeds of South China were analyzed with the help of single nucleotide polymorphism (SNP) chips. The results showed that the genetic diversity of South China indigenous pigs is declining rapidly, and gene introgression from commercial pigs to indigenous pigs was detected. Selection signature analysis showed differences among South China indigenous pig breeds, commercial pig breeds, and wild pig breeds were present for meat quality and growth. Our study deepened understanding of the conservation status and selection mechanisms of Chinese indigenous pigs.

**Abstract:**

To investigate the genetic diversity, population structure, extent of linkage disequilibrium (LD), effective population size (Ne), and selection signatures in indigenous pigs from Guangdong and Guangxi in China, 226 pigs belonging to ten diverse populations were genotyped using single nucleotide polymorphism (SNP) chips. The genetic divergence between Chinese and Western pigs was determined based on the SNP chip data. Low genetic diversity of Dahuabai (DHB), Luchuan (LC), Lantang (LT), and Meihua (MH) pigs, and introgression of Western pigs into Longlin (LL), MH, and Yuedonghei (YDH) pigs were detected. Analysis of the extent of LD showed that indigenous pigs had low LD when pairwise SNP distance was short and high LD when pairwise SNP distance was long. Effective population size analysis showed a rapid decrease for Chinese indigenous pigs, and some pig populations had a relatively small Ne. This result indicated the loss of genetic diversity in indigenous pigs, and introgression from Western commercial pigs. Selection signatures detected in this study overlapped with meat quality traits, such as drip loss, intramuscular fat content, meat color b*, and average backfat thickness. Our study deepened understanding of the conservation status and domestication of Chinese indigenous pigs.

## 1. Introduction

The pig is one of the most important livestock animals, providing the majority of protein for humans [1]. In China, the slaughtering capacity of pigs reaches 0.650 billion every year and pork accounts for about 52% of the meat consumption of Chinese citizens. China is also rich in pig breeding resources: China is reported to have up to 100 pig breeds, making up about one-third of global pig breeds [2]. Chinese indigenous pigs have many desirable characteristics such as high reproduction, high intramuscular fat content, disease-resistance ability, and strong resistance to extreme climates [3]. Chinese indigenous pig breeds have been used for meat production for thousands of years in China. However, with the introduction and popularization of Western commercial pig breeds, the populations of indigenous pigs have rapidly decreased. Many Chinese indigenous pig breeds are only preserved in a few reservation farms with small populations, and some pig breeds are even reported to be extinct [4].

Diversity in animal breeding resources is an important component of biodiversity and is the basis of genetic improvement in domesticated animals. Although some Chinese indigenous pigs have low growth rates and low feed conversion efficiency, desirable traits such as early puberty and good meat quality should not be underrated. Chinese pig breeds could be used to produce new breeds with good meat quality or high reproduction. Furthermore, the migration and evolution of pigs has always been closely linked with human migration, and understanding pig evolution is thus significant for understanding human migration and culture. Weitzman [5] suggested it is important to quantify biodiversity to better rationalize conservation policies. Caballero and Toro [6] reported the ideal conservation management is minimizing the variances of contributions from ancestors to descendants in all previous generations.

Many studies have been conducted on the genetic diversity and evolution of Chinese indigenous pigs based on genetic markers such as restriction fragment length polymorphism, simple sequence repeats, or mitochondrial DNA [7,8]. It was thought that Chinese indigenous pigs have high genetic diversity compared with Western pigs and that Chinese indigenous pigs and Western pigs originated independently from European and Asian wild pigs, respectively. However, the Western commercial pig has genes from Chinese indigenous pigs, which indicates contributions from Chinese indigenous pigs in Western commercial pig cultivation [9,10,11]. Many Chinese pigs have also experienced genetic introgression from Western commercial pigs due to crossbreeding [4]. It is important to detect the genetic diversity and population structure of the indigenous pig breeds to better protect them.

Some studies of selection signatures have focused on China’s indigenous pig breeds. A study based on Laiwu pigs and Yorkshire pigs reported 175 potential selection regions, covering 43.750 Mb of genomic regions. Genes from the potential selection regions involved in feed intake, fat deposition, and immune response were annotated [12]. The adaptive haplotype in the northern Chinese populations was likely introduced from another divergent *Sus* species as reported by Linah et al. [13]. Moreover, the unique domestication of Meishan pigs that occurred in the Taihu Basin area between the Majiabang and Liangzhu cultures resulted in the positive selection of 300 protein-coding genes as reported by Zhao et al. [14].

Studies of genetic diversity in Chinese indigenous pig breeds are almost all based on a small number of genetic markers and there are few systematic studies on indigenous pigs of the Guangdong and Guangxi provinces. This study therefore investigated genetic diversity, population structure, linkage disequilibrium (LD), and effective population size (Ne) within or between 11 pig breeds in the Guangdong and Guangxi provinces using genome-wide single nucleotide polymorphisms (SNPs) generated from the SNP BeadChip.

## 2. Materials and Methods

### 2.1. Ethics Statement

Animal care and all experiments were conducted according to the Regulations for the Administration of Affairs Concerning Experimental Animals (Ministry of Science and Technology, China, revised June 2004) and approved by the Animal Care and Use Committee of the South China Agricultural University, Guangzhou, China (approval number SCAU#2013-10).

### 2.2. Sample and Data

A total of 825 animals including ten indigenous pig breeds, four Western commercial pig breeds, and European and Asian wild pig breeds were used in this study. Throughout the manuscript, we use “Western pigs” to refer to Western commercial pig breeds and European wild pig, whereas “Chinese pigs” refers to indigenous pig breeds and the Asian wild pig. Indigenous pig breed samples were collected from the Guangdong and Guangxi provinces of South China with sample sizes ranging from 15 to 34 (Table 1). Related animals or those showing evidence of hybridization with other breeds were avoided when possible. Ear tissue samples were fixed in 95% alcohol and stored in −20 °C freezers. Genomic DNA was extracted from ear tissue using the E.Z.N.A.^®^Tissue DNA Kit (D3396-02, Omega Bio-tek, Norcross, GA, USA) according to the manufacturer’s instructions.

Among the indigenous pigs, samples of Guizhonghua (GZH), Bamaixnag (BMX), Dahuabai (DHB), Meihua (MH), Lantang (LT), and Yuedonghei (YDH) were genotyped with the Illumina Porcine SNP60 v1 BeadChip, which contains 62,163 SNPs; Longlin (LL), Debao (DB), Luchuan (LC), and Guangdongxiaohua (XE) were genotyped using the GeneSeek-Neogen PorcineSNP80 BeadChip (Neogen Corporation, Lansing, MI, USA) which contains 68,528 SNPs. The genomic SNP data of the Duroc (DR), Landrace (LR), Large White (LW), Pietrain (PI), European wild boar (WBE) and Asian wild boar (WBA) was available in the datadryad database (https://datadryad.org/); these breeds were genotyped using Illumina Porcine SNP60 v1 or v2 BeadChip (Illumina, San Diego, CA, USA). We used data downloaded from the database directly, for details of these data please refer to Yang et al. [15].

PLINK [16] software was used for data combination and quality control. A total of 41,344 SNPs common to all animals were extracted. The SNPs with a call rate < 0.090 and minor allele frequency (MAF) < 0.050 were removed, and individuals with SNP call rates < 0.900 were also removed. After quality control, a total of 41,217 SNPs and 818 individuals were retained for further analysis. Among the SNPs that passed the quality control, 34,709 were unambiguously mapped to the pig genome and 6508 could not be unambiguously mapped. The SNP data of 818 animals are available in the figshare database (https://figshare.com/s/459f0a85cba4f694d8f8).

### 2.3. Genetic Diversity Analysis

In order to detect the genetic diversity of each breed, five parameters including allelic richness (A_R_), the number of SNPs with MAF ≥ 0.200 (N_SNP_), the proportion of polymorphic markers (P_N_), observed heterozygosity (H_O_), and expected heterozygosity (H_E_) were calculated. In order to remove the bias caused by the SNP chips, which were designed without data from the Chinese pigs, the common subset of SNPs with MAF ≥ 0.200 in both Western pigs and Chinese pigs was selected. P_N_, H_O_, and H_E_ were calculated using PLINK; A_R_ was calculated using ADZE [17] software.

### 2.4. Phylogenetic Analysis

Pairwise genetic distances between all individuals (*D*) were estimated as one minus the average proportion of alleles shared, which was calculated as $D_{st}$ using PLINK [16]:

$$D_{st}=\frac{IBS2+0.5\times IBS1}{N}$$

where IBS1 and IBS2 refer to the number of loci that two individuals share, either 1 or 2 alleles identical by state, respectively, and N is the total number of loci; all 41,217 SNPs were used in this process.

Pairwise genetic distance among all pig breeds was estimated using Nei’s standardized genetic distance [18] $D_{xy}$:

$$D_{xy}=-In\left[\frac{\sum_{i=1}^{n}\sum_{j=1}^{ki}x_{ij}y_{ij}}{\sqrt{\left(\sum_{i=1}^{n}\sum_{j=1}^{ki}x_{ij}^{2}\right)\left(\sum_{i=1}^{n}\sum_{j=1}^{ki}y_{ij}^{2}\right)}}\right].$$

In the above formula, $x_{ij}$ and $y_{ij}$ are the allele frequencies of the jth allele at the ith loci of groups x and y, respectively, ki and n are the number of alleles at the ith locus and the number of loci, respectively; all 41,217 SNPs were used.

Two phylogenetic trees based on the pairwise genetic distances between individuals and the Nei’s standardized genetic distance were constructed using the neighbor-joining method in MEGA software [19].

### 2.5. Population Structure Analysis

To investigate the population structure of the pig breeds in this study, principal component analysis (PCA) and structure analysis were conducted. GCTA [20] software was used for PCA and all 34,709 autosomal SNPs that passed the quality control check were used. STRUCTURE software [21] was used to estimate ancestral relationships among all breeds. In order to avoid artifacts due to LD, and reduce the running time, only SNPs with pairwise genotypes $r^{2}\leq 0.200$ and MAF ≥ 0.200 chosen by PLINK [16] were used. The analysis was performed for 2–17 clusters among all breeds and 2–11 clusters among South China indigenous breeds using the default settings.

### 2.6. Linkage Disequilibrium and Effective Population Size Analysis

The extent of LD was calculated using the genotype correlation coefficient ($r^{2}$) within each breed; autosomal SNPs with MAF ≥ 0.050 and call rate ≥ 0.900 were used. LD was calculated for all pairs of SNPs with distances within 20 Mb using SNeP software [22]. The LD calculation formula was as follows:

$$r_{X,Y}^{2}=\frac{{\left[\sum_{i=1}^{n}\left(X_{i}-\bar{X})(Y_{i}-\bar{Y}\right)\right]}^{2}}{\sum_{i=1}^{n}{\left(X_{i}-\bar{X}\right)}^{2}\sum_{i=1}^{n}{\left(Y_{i}-\bar{Y}\right)}^{2}}$$

where $\bar{X}$ and $\bar{Y}$ are the mean genotype frequencies for the first and second locus respectively, $X_{i}$ is the genotype of individual i at the first locus and $Y_{i}$ is the genotype of individual i at the second locus, and n is the number of individuals. Pairs of SNPs were divided into 100 bins according to their pairwise physical distance; pairs of SNPs with intervals from 0 to 0.200 Mb were the first group, from 0.200 to 0.400 Mb were the second group, and so on, with a maximal distance of 20 Mb. Observed LD ($r^{2}$) was averaged for distance bins for each SNP pair. A non-linear model was used to fit the relationship between $r^{2}$ and physical distance:

$$LD=\frac{a-d}{1+{\left(\frac{dist}{c}\right)}^{b}}+d+e$$

where LD was the observed LD for each marker pair with the SNP distance of dist, which was the average SNP distance of distance bins for each SNP pair; a, b, c, and d were the four parameters of this model and e was the random residual.

The Ne of each breed was estimated based on the LD using the follow equation [23]:

$$N_{e(t)}=\frac{1}{\left(k\times m\right)}\left(\frac{1}{E\left[r^{2}\right]}-\alpha \right)$$

where $N_{e(t)}$ was the ancestral Ne of t generations ago, $r^{2}$ was estimated by the LD decline model, and m was the recombination frequency in Morgan’s estimated by the equation $m=dist*(1-\frac{dist}{2})$ [24], dist was the physical distance of pairs of SNPs in 100 Mb, the relationship between m and t was ${m=(2t)}^{-1}$, meaning that the LD of ct can be used to estimate the Ne t generations ago. The ancestral Ne of 3 to 100 generations were estimated for each breed. The LD decline analysis and ancestral Ne estimation were conducted using programs that we developed in R [25].

### 2.7. Genetic Differentiation and Detection of Selection Signatures

To further investigate the population differentiation of indigenous pigs of South China, Western commercial pig breeds, and European and Asian wild pig breeds the *F_ST_* statistic was calculated using the Genepop R package [26]. The formula for *F_ST_* was:

$$F_{ST}=\frac{MSP-MSG}{MSP+\left(n_{C}-1\right)MSG}$$

where MSP and MSG denote the observed mean square errors for loci between and within populations, respectively, and $n_{C}$ is the average sample size across samples that also incorporates and corrects for the variance in sample size over the population. More details are shown in Su et al. [27]. The top 1% of SNPs were defined as significant [28].

### 2.8. Genome Annotation and Quantitative Trait Loci Overlapping with Potential Selection Signatures

The 200 kb upstream and downstream of significant SNPs were defined as potential selection regions. Genome annotations were based on *Sus scrofa* genome 11.1 (https://www.animalgenome.org/blast/). Genes overlapping with potential selection regions were treated as candidate genes. Both RNA and unconfirmed genes were filtered out. The Animal Quantitative Trait Loci (QTL) Database [29] was used to explore the QTL that overlapped with the potential selection regions.

### 2.9. Enrichment Analysis

To understand the biological functions of the candidate genes, the Database for Annotation, Visualization, and Integrated Discovery (DAVID) Version 6.8 (https://david.ncifcrf.gov/) [30,31] was used for enrichment analysis. *p*-values of enriched Kyoto Encyclopedia of Genes and Genomes (KEGG) pathways [32] and Gene Ontology (GO) [33] biological processes below 0.05 were treated as significant in this study.

## 3. Results

### 3.1. Genetic Diversity Analysis

There were 31,253 SNPs with MAF ≥ 0.200 in Western pigs and 18,306 SNPs in Chinese pigs. Only 12,808 SNPs with MAF ≥ 0.200 were found in both Western pigs and Chinese pigs; these SNPs were used in genetic diversity analysis. The MAF distribution of the 12,808 SNPs is shown in Figure S1.

For P_N_, the Western pigs had a comparable level of polymorphism with a P_N_ range from 0.993 to 1, compared with Chinese pigs with a P_N_ range from 0.984 to 1. DB, LC, LL, and XE had slightly lower P_N_ compared with the other South China pig breeds.

For N_SNP_, in Western pigs, LR had the highest N_SNP_ value (N_SNP_ = 10,483) and WBW had the lowest (N_SNP_ = 7893). In Chinese pigs, LL had the highest N_SNP_ value (N_SNP_ = 11,008) and DHB had the lowest (N_SNP_ = 7047). Overall, the Western commercial pigs had the highest P_N_ while the Chinese pigs had the lowest and the wild boar was in the middle. For heterozygosity, the Chinese pigs and Western pigs had comparable H_E_ and H_O_, with H_O_ ranging from 0.251 to 0.432 and H_E_ ranging from 0.311 to 0.420. YDH had the highest H_O_ and WBE had the lowest. LL had the highest H_E_ and DHB had the lowest. It was also observed that all H_E_ of the commercial pigs and wild boars were far below their H_O_, but the opposite results occurred in Chinese pigs. All breeds had almost the same A_R_, which ranged from 1.898 to 1.998. More details of the genetic diversity of each breed are shown in Table 1.

### 3.2. Genetic Distance Analysis

**Figure 1:**
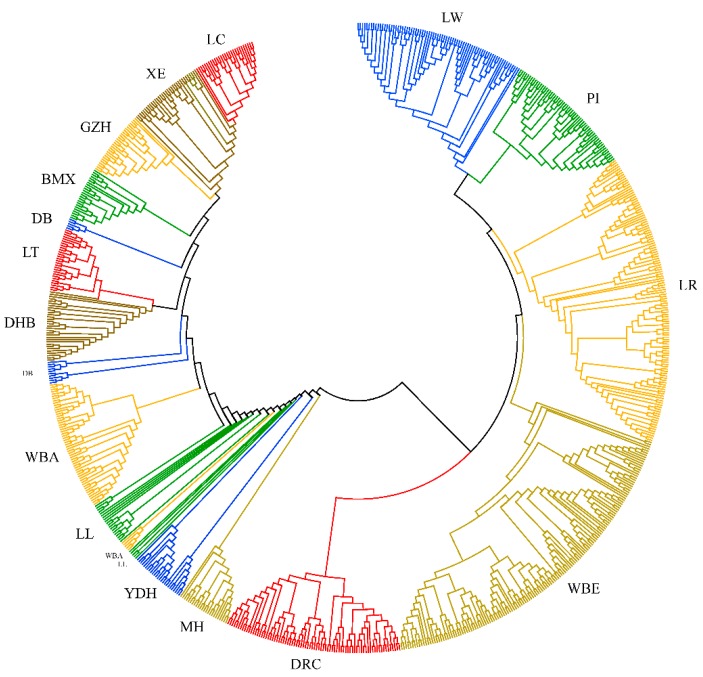
The neighbor-joining tree of the tested populations ^1^. ^1^ DR = Duroc, LW = Large White, LR = Landrace, PI = Pietrain, WBE = European wild boar, WBA = Asian wild boar, BMX = Bamaxiang, DB = Debao, DHB = Dahuabai, GZH = Guizhonghua, LC = Luchuan, LL = Longlin, LT = Lantang, MH = Meihua, XE = Guangdongxiaohua, YDH = Yuedonghei.

The average genetic distance between pairs of individuals (D) was 0.366 ± 0.061 within Western pigs, 0.233 ± 0.048 within Chinese pigs, and 0.429 ± 0.070 between Western and Chinese pigs (Figure S2). Within each breed, the average genetic distance ranged from 0.128 (LC) to 0.251 (LL) in Chinese pigs, and from 0.244 (WBE) to 0.309 (LW) in Western pigs. A phylogenetic tree was constructed based on D (Figure 1). Individuals of the same breed tended to cluster together in a distinct branch except for LL, YDH, DB, and WBA. The phylogenetic tree was roughly divided into two branches. The four Western commercial pigs and European wild boar clustered together in a big branch and the ten South China indigenous pigs and Asian wild boar clustered together in the other branch. Among the indigenous pigs, LL, YDH, and MH clustered closer to the Western pigs compared with the other indigenous pigs. Some breeds, such as DRC and WBE, were further divided into several smaller branches. The genetic distance of each individual is shown in Table S1.

**Figure 2:**
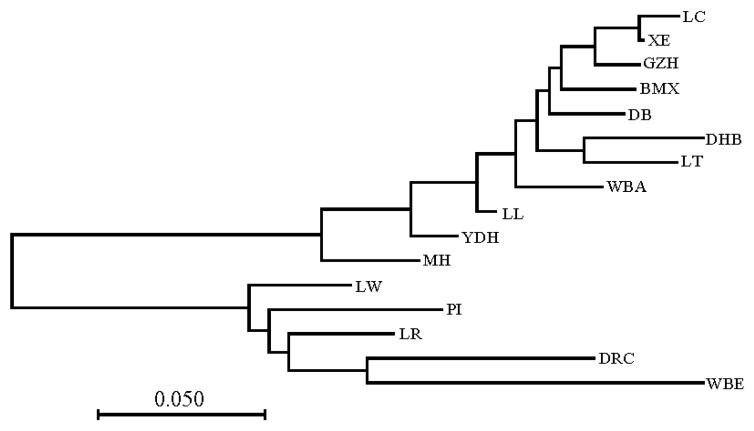
The neighbor-joining tree based on Nei’s genetic distance ^1^. ^1^ DR = Duroc, LW = Large White, LR = Landrace, PI = Pietrain, WBE = European wild boar, WBA = Asian wild boar, BMX = Bamaxiang, DB = Debao, DHB = Dahuabai, GZH = Guizhonghua, LC = Luchuan, LL = Longlin, LT = Lantang, MH = Meihua, XE = Guangdongxiaohua, YDH = Yuedonghei.

Nei’s standard genetic distance ($D_{xy}$) was used to measure the genetic distance of each pair of breeds. The $D_{xy}$ ranged from 0.013 (LC vs. XE) to 0.471 (LC vs. WBE). The phylogenetic tree constructed based on $D_{xy}$ is shown in Figure 2. The tree was roughly divided into two branches, the same as the tree constructed based on D. The $D_{xy}$ between Chinese pigs was smaller than that between Western pigs. MH, YDH, and LL were closer to the Western pigs than other indigenous pigs.

### 3.3. Population Structure Analysis

#### 3.3.1. Principal Component Analysis-Based Clustering

**Figure 3:**
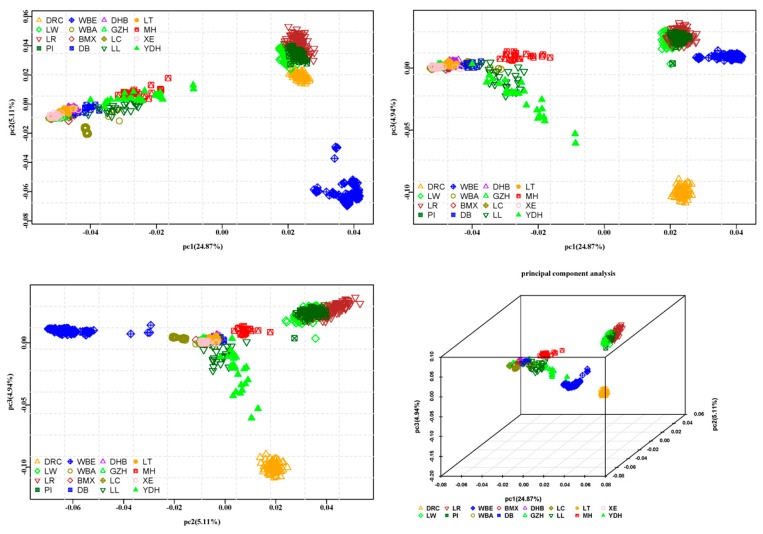
Principal component analysis results ^1^. ^1^ DR = Duroc, LW = Large White, LR = Landrace, PI = Pietrain, WBE = European wild boar, WBA = Asian wild boar, BMX = Bamaxiang, DB = Debao, DHB = Dahuabai, GZH = Guizhonghua, LC = Luchuan, LL = Longlin, LT = Lantang, MH = Meihua, XE = Guangdongxiaohua, YDH = Yuedonghei.

PCA was used in cluster analysis based on the genotype of each individual (Figure 3). The contributions of the first three principal components (pc) were 24.87%, 5.11%, and 4.94% respectively, and the accumulated contributions of the first ten principal components were 44.19%. Discrimination of Chinese pigs from Western pigs was clear on pc1, and WBE was separated from Western commercial pigs on pc2. Duroc was separated from PI, LW, LR, and WBE. In general, the first three pc divided all breeds into four clusters, WBE and DRC were two clusters, the Chinese pigs formed another cluster, and the remaining breeds (PI, LW, and LR) formed the last cluster. The PCA results were consistent with the neighbor-joining tree. In addition, PCA was used in cluster analysis based on the genotype of each South China indigenous individual (Figure S3). The MH was separated from other South China indigenous pig breeds. Furthermore, the first three pc divided ten South China indigenous breeds into four clusters, MH and LL were two clusters, DHB and LT formed another together, and the remaining South China indigenous breeds formed the last cluster (LL, DB, BMX, GZH, LC, and XE).

#### 3.3.2. Population Cluster Based on STRUCTURE

**Figure 4:**
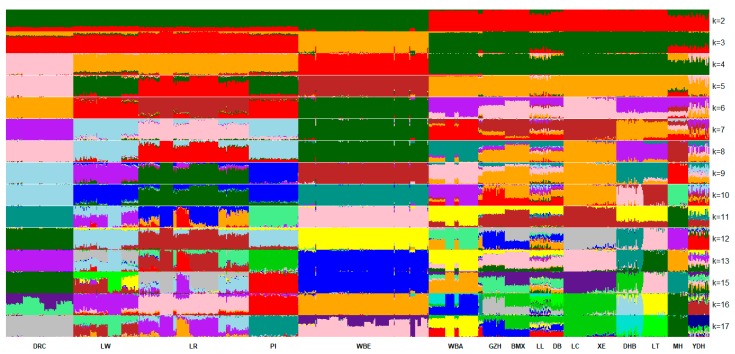
Structure results ^1^. ^1^ DR = Duroc, LW = Large White, LR = Landrace, PI = Pietrain, WBE = European wild boars, WBA = Asian wild boars, BMX = Bamaxiang, DB = Debao, DHB = Dahuabai, GZH = Guizhonghua, LC = Luchuan, LL = Longlin, LT = Lantang, MH = Meihua, XE = Guangdongxiaohua, YDH = Yuedonghei.

A cluster analysis based on the Markov chain model using the genotype data was implemented in STRUCTURE software [21]. To reduce the calculation burden, 18,946 SNPs with pairwise genotypes $r^{2}\leq 0.2000$ and MAF ≥ 0.200 were used. We analyzed the grouping situation when K ranged from 2 to 17, meaning that we presupposed that all individuals originated from K ancestors or breeds. The likelihood of each K value is shown in Figure S4; the likelihood was the highest when K = 17, indicating that 17 may be the most suitable number of ancestries. The cluster results based on STRUCTURE are shown in Figure 4. When K = 2, the Western pigs and Chinese pigs had two distinct ancestries; MH, LL, and YDH had large proportions of ancestry of Western pigs. The four commercial pig breeds had large proportions of ancestry that were the same as Chinese pigs; WBE lacked any affinity with Chinese pigs. European wild boars were separated from commercial pigs when K = 3; DRC separated from LW, LR, and PI when K = 4; and LR separated from LW and PI when K = 5. The Chinese pigs gradually separated from each other when K ranged from 6 to 17. When K = 17, the ancestry of LC and XE was almost the same. It was clear that GZH, BMX, LL, DB, LC, and XE share a large proportion of ancestry; BMX seemed to be a hybrid of XE (LC) and GZH. Taken together, the STRUCTURE results showed that MH and YDH experienced introgression from LW and DRC, respectively, and the Chinese pigs shared some ancestry. The population clusters based on STRUCTURE analysis of South China indigenous pig breeds are shown in Figure S5. The likelihood of each K value is shown in Figure S6; the likelihood was the highest and the standard deviation was smallest when K = 9, indicating that 9 may be the most suitable number of ancestries among South China indigenous pig breeds.

### 3.4. Linkage Disequilibrium and Analysis of Effective Population Size

**Figure 5:**
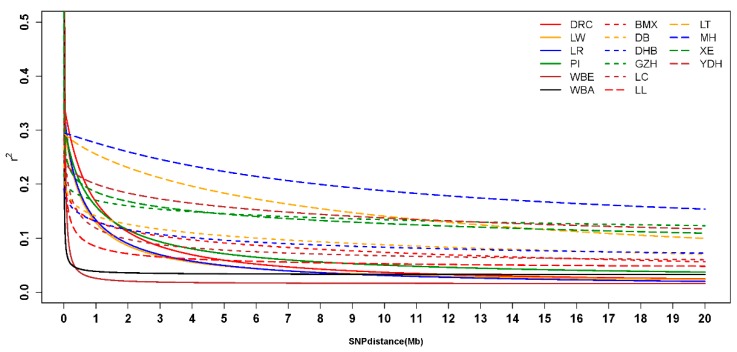
Extent of linkage disequilibrium (LD) for each breed ^1^. ^1^ DR = Duroc, LW = Large White, LR = Landrace, PI = Pietrain, WBE = European wild boars, WBA = Asian wild boar, BMX = Bamaxiang, DB = Debao, DHB = Dahuabai, GZH = Guizhonghua, LC = Luchuan, LL = Longlin, LT = Lantang, MH = Meihua, XE = Guangdongxiaohua, YDH = Yuedonghei.

Considering the absence of a known phase of all individuals, squared Pearson’s product-moment correlation coefficients between pairs of SNPs were used to measure the LD. Average $r^{2}$ at various distances was computed by grouping all SNP pairs by their pairwise physical distance in classes of 200 kb of length, starting at 0 to 20 Mb. A non-linear model was used to fit the LD decrease of each breed. The model-fitting degrees of all breeds were high, reaching 0.950 (Table S2). The predicted $r^{2}$ of different distances and the true $r^{2}$ are shown in Figure S7, indicating that the non-linear model was a good fit for the LD decline. LD declined with increasing distance between SNP pairs, as shown in Figure 5. The $r^{2}$ of WBE, WBA, and DHB declined rapidly, while that of MH declined slowly. The $r^{2}$ of WBE was the lowest, followed by WBA, and MH had the highest among all breeds overall. The $r^{2}$ of commercial pigs was higher than almost all the South China indigenous pigs when the distance was short, while over long distances this result was reversed.

**Figure 6:**
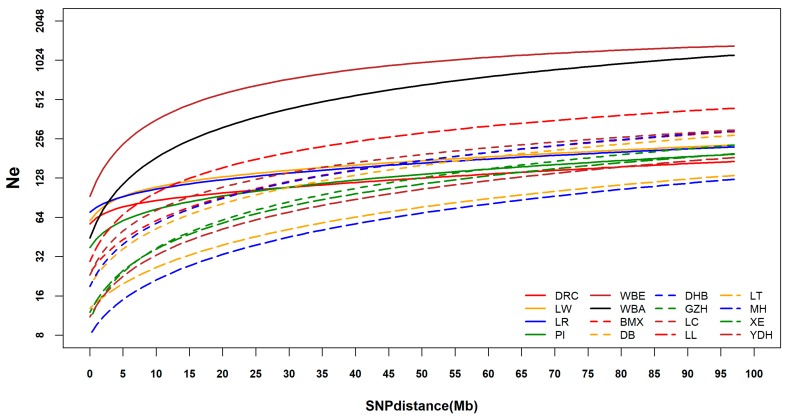
Historical effective population size (Ne) of each breed ^1^. ^1^ DR = Duroc; LW = Large White; LR = Landrace; PI = Pietrain; WBE = European wild boar; WBA = Asian wild boar; BMX = Bamaxiang; DB = Debao; DHB = Dahuabai; GZH = Guizhonghua; LC = Luchuan; LL = Longlin; LT = Lantang; MH = Meihua; XE = Guangdongxiaohua; YDH = Yuedonghei.

The historical Ne from 100 generations to 3 generations ago of each breed were estimated based on the LD decay. Declines at different speeds were observed in the Ne of all breeds from distant generations to recent generations (Figure 6). About six generations ago, the Ne of WBE and WBA were larger than those of the domestic pigs. From six generations ago to three generations ago, the Ne of WBA dropped rapidly and became smaller than that of several domestic pigs. LL had the largest Ne among the domestic pigs, but about 15 generations ago, its Ne gradually became smaller than those of LW and LR. Overall, the wild boars had large Ne, and the Chinese pigs had large Ne many generations ago compared to the Western pigs but had smaller Ne in recent generations. Three generations ago, WBE had the largest Ne (Ne = 150.530) among all breeds and LT had the smallest Ne (Ne = 15.700). Among the Western commercial pigs, PI had the smallest Ne (Ne = 48.760) and LR had the largest (Ne = 81.230). In Chinese indigenous pigs, the Ne of LL was the largest (Ne = 45.410), MH had the smallest Ne (Ne = 11.020).

### 3.5. Genetic Differentiation, Outlier Loci, and Candidate Genes under Selection

**Figure 7:**
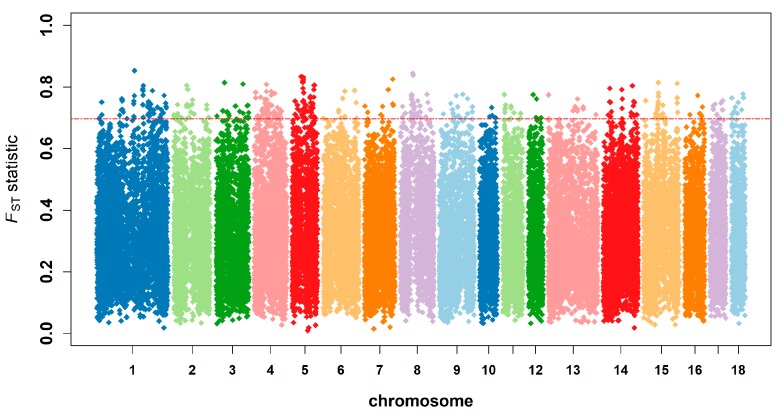
Genome-wide distribution of selection signatures detected by *F_ST_* statistics across all autosomes in South China pig breeds, Western commercial pig breeds, and European and Asian wild boar. The SNPs above the red line were the significant SNPs detected in this study.

The *F_ST_* value of indigenous pigs of South China, Western commercial pig breeds, and European and Asian wild pig populations ranged from 0.010 to 0.852 (with an average value of 0.316). A total of 347 SNPs were treated as significant (*F_ST_* statistic > 0.697) in this study (Figure 7). Among these potential selection regions, the highest distribution was observed on *Sus scrofa* chromosome (SSC)1, SSC4, and SSC5, representing 40.630% of the potential selection regions [(50+46+45÷347)]. The top six potential selection regions are shown in Table 2. All potential selection regions are shown in Table S3 and the *F_ST_* of all SNPs are shown in Table S4.

### 3.6. Genome Annotation and Quantitative Trait Loci Overlapping with Potential Selection Signatures

A total of 895 candidate genes were enriched in 347 potential selection regions. Candidate genes are shown in Table 2 and Table S3. QTLs overlapping with these potential selection regions were associated with some meat quality traits such as drip loss, intramuscular fat content, meat color b*, and average backfat thickness (Table 2 and Table S3). The average daily gain trait was the most reported in potential selection regions.

### 3.7. Enrichment Analysis

Twenty GO biological processes were enriched in this study, the most significant GO term was GO: 0050482-arachidonic acid secretion (*p*-value: 0.002). The GO: 0045944-positive regulation of transcription from RNA polymerase II promoter (*p*-value: 0.021) was enriched in most candidate genes (count: 29) in this study (Table 3 and Table S5). In addition, eight KEGG pathways were enriched in this study; ssc00592: alpha-Linolenic acid metabolism (*p*-value: 0.001) was the most significant. The top five KEGG pathways and GO biological processes are shown in Table 3. More details of KEGG pathways and GO biological processes are shown in Table S5.

## 4. Discussion

### 4.1. Genetic Diversity of Indigenous Pigs

We assessed the genetic diversity of indigenous pigs from Guangdong and Guangxi provinces using the SNP chips; four Western commercial breeds and two wild pig breeds were included. The SNP chips were primarily designed based on the genomes of several Western pigs with wild boar as a reference [34] and did not include data from Chinese indigenous pigs. Therefore, the chips were biased such that we expected SNPs from Western pigs to show more diversity. In this study, 31,253 SNPs with MAF ≥ 0.200 were found in Western pigs, but only 18,306 SNPs were found in Chinese pigs. To reduce the bias caused by the SNP chips design, the common SNPs between Western and Chinese pigs were used in genetic diversity analysis. The Western commercial pigs experienced intensive selection, meaning it is reasonable to think they would have lower genetic diversity compared with Chinese indigenous pigs. This hypothesis has been verified in many previous studies [35]. In this study, the Western commercial pigs had genetic diversity comparable to that of the Chinese pigs. The genetic diversity comparison between Chinese pigs and within Western pigs may be less useful because of the SNP chips design. However, comparison within Chinese pigs or within Western pigs is likely to be reliable.

Among the four commercial pig breeds, LW, LR, and PI had comparable genetic diversity. The genetic diversity of DRC was slightly lower, which was also found in other studies [36,37]. DHB, LT, MH, and LC had low values for each genetic diversity parameter, which was consistent with the conservation status of these pigs. LL and YDH had extremely high genetic diversity.

It was also observed that the H_E_ values of all commercial pigs and wild boars were far below their H_O_, but in Chinese pigs the opposite result was found. This phenomenon may have occurred because: (1) the samples of commercial pigs and wild boars came from multiple sources, while only a single resource was used for the samples from Chinese indigenous pigs (the samples of each Chinese indigenous pig breed were from the same population); or (2), the bias caused by the SNP chips. Although the common subset of SNPs with MAF ≥ 0.200 in both Western pigs and Chinese pigs was selected, a number of SNPs with MAF < 0.200 were found in each South China indigenous pig breed. This phenomenon may have occurred because the MAF distribution of 12,808 was not the same in each breed. The low H_O_ value and large difference between H_O_ and H_E_ may indicate fragmentation of the wild boars’ habitat.

### 4.2. Genetic Distance and Population Structure Analysis

In this study, one minus the average proportion of alleles shared by pair-wise individuals was treated as the genetic distance between two individuals. The phylogenetic tree constructed based on individual genetic distances showed that individuals from the same breed usually grouped together. The Chinese indigenous pigs clustered together with WBA, and the Western commercial pigs and WBE clustered in the other branch. This result indicates that the Chinese indigenous and Western commercial pigs originated from WBA and WBE, respectively. The clustering of LL and YDH was loose, and together with MH they were distributed between Western pigs and Chinese indigenous pigs. The phylogenetic tree based on Nei’s genetic distance also indicated that LL, MH, and YDH had a close genetic distance with Western pigs compared with the other Chinese indigenous pigs. The clustering of the PCA analysis showed the same results as the phylogenetic analysis. These results indicated that all of the Chinese pig breeds may have undergone hybridization with Western pigs.

The PCA analysis divided the ten South China indigenous breeds into four clusters (Figure S3). Except for LL and MH, other two clusters were consistent with the sample site (LT and DHB were sampled from Guangdong province, LL, DB, BMX, GZH, LC were sampled from Guangxi province). Although XE was sampled from Guangdong province, the XE and LC were subpopulations of the Liangguangxiaohua pig [2]. This could refer to the clustering of PCA analysis based on ten South China indigenous breeds related to the geographical isolation of the sampling site.

The population structure detected using STRUCTURE showed that when K = 2, the Western commercial pigs shared almost the same lineage as the WBE except that they only shared a small proportion of lineage with Chinese pigs. When K = 3, WBE appeared to have no relationship with the Western commercial pigs and Chinese pigs; the Western commercial pigs and Chinese pigs had few common lineages. These results indicated that the Western commercial pigs and Chinese indigenous pigs independently originated from WBE and WBA, respectively. The lineages of LL, MH, and YDH seemed to mix and shared more lineage with Western commercial pigs compared with other Chinese indigenous pigs. The LL and DB showed similar results for population structure in Figure 4, however, in Figure S5, the population structures of LL and DB were different. This phenomenon may have occurred because only South China indigenous pig breeds were analyzed in Figure S5, and it could be easier to distinguish them than when analyzed with all pig breeds. This indicated introgression from Western commercial pigs and provided evidence that LL and YDH hybridized with DRC, and MH hybridized with LR. The lineages of LC and XE were almost the same, consistent with the fact that LC and XE are the same breed in traditional classification. GZH and BMX had a large proportion of common lineages due to close production regions leading to high gene flow.

### 4.3. Linkage Disequilibrium and Effective Population Size Analysis

LD refers to nonrandom association of alleles at different loci and is the basis of genetic mapping and genomic selection. LD information can also be used to estimate the Ne and reveal the evolutionary history of breeds. In this study, because of the small population size of many breeds and the unknown phase of the genotype, the genotype correlation coefficient was used as a measurement of the extent of LD.

Wild boars had low LD compared with domestic pigs. During the domestication of pigs, only some wild boars were successfully domesticated. Thus, Ne gradually decreased, inbreeding gradually increased, and consequently the LD increased. Among the Western commercial pigs, the LD of DRC was the highest, which was in accordance with other research [38,39]. The Chinese indigenous pigs had much lower LD compared with Western commercial pigs when the physical distance between pairwise SNPs was small, while larger distances led to the opposite result. The Ne analysis showed that the Chinese indigenous pigs from many generations ago had higher Ne than Western commercial pigs, while three generations ago, most of the Chinese indigenous pigs had lower Ne than Western commercial pigs. The low Ne of LL and YDH, combined with high genetic diversity of LL and YDH and close genetic distance with Western pigs fully proved that LL and YDH experienced introgression from Western commercial pigs.

### 4.4. Genetic Differentiation and Detection of Selection Signatures

The average *F_ST_* of indigenous pigs of South China, Western commercial pig breeds, and European and Asian wild pig populations was 0.316. After comparing Laiwu pigs and Yorkshire pigs, Chen et al. [12] found that the average *F_ST_* was 0.169. The degree of genetic differentiation among four populations in our study was higher than this result. The reason for this might be related to the genetic structure of pig populations used in this study. The commercial pig breeds have experienced stronger artificial selection than South China indigenous pigs. The biological function of potential selection regions in this study show the same result as Wang et al. [40] reported: the common goal of domestication breeding in China and Europe was mostly the same. However, Chinese breeding has focused on fat deposits and reproduction, while European breeding tends to prefer leanness and modifying body length.

The candidate gene *CYB5A* annotated in top 6 potential selection regions was once reported that European pig breeds and Licha black pig breeds were dominated by haplotype A (G-T-C), whereas other groups of Chinese breeds and wild boar were dominated by the haplotype B (delG-C-T). This variation of the porcine *CYB5A* promoter region might explain the differences in androstenone accumulation between Chinese and European pig breeds [41] and could indicate the results of this study are reliable. Another candidate gene, *HPOX*, annotated in the top six potential selection regions, was implicated in a report that chromatin-modifying enzyme histone deacetylase 2 (Hdac2), *HPOX* and Gata4 coordinately cardiac myocyte proliferation during embryonic development. It can be inferred that *HPOX* is related to embryonic development. In addition, three candidate genes (*MC1R*, *MC2R*, and *MC5R*) are members of the melanocortin receptor gene family. *MC1R* is associated with the coat color of Duroc [42,43], and *MC5R* influences the physiological color change with *MC1R* in flounder [44]. It can be inferred that the three candidate genes may result in the differences of coat color among the four populations.

The most significant KEGG pathway was ssc00592: alpha-Linolenic acid metabolism (*p*-value: 0.001). The alpha-Linolenic acid could improve development of the mammalian embryo in vitro, and it could enhance the reproductive performance of pigs [45]. This KEGG pathway may related to the high fertility of South China indigenous pig breeds. The pathway of GO: 0048706-embryonic skeletal system development (*p*-value: 0.029, shown in Table S5) in this study. Skeletal muscle tissue is a significant contributor to birth weight birth weight influences the survival rate as Milligan et al. [46] reported. This result may relate to the selection of reproduction in the pig domestication process.

A number of QTLs associated with meat quality such as drip loss, intramuscular fat content, meat color b*, and average backfat thickness, overlapped with the potential selection regions. Chinese indigenous pigs are famous for superior meat quality and the result in which QTL overlapped with the potential selection regions may explain the genetic variation between Chinese indigenous pig breeds and commercial pig breeds.

## 5. Conclusions

In conclusion, a comprehensive analysis of genetic diversity, population genetic structure, LD, Ne, and the selection signatures of ten indigenous pig breeds (groups) distributed in Guangdong and Guangxi was performed using SNP chips. The rapid decline of genetic diversity, effective population size of indigenous pigs and gene introgression from commercial pigs (LW and DRC) to indigenous pigs (YDH and MH) were detected. Differences among South China indigenous pig breeds, commercial pig breeds, and wild pig breeds were present for meat quality and growth. Our study deepened the understanding of the conservation status and selection mechanisms of Chinese indigenous pigs.

## Figures and Tables

**Table 1:** Sample size and genetic diversity of each breed ^1^.

Breed ^1^	Origin	No.	Sex Composition, Male/Female/Ambiguous	N_SNP_^2^	P_N_^3^	H_O_^4^	H_E_^5^	A_R_^6^	Ne ^7^
DRC	-	79	8/12/59	8010	0.998	0.305	0.334	1.940	67.920
LW	-	76	8/12/56	10,135	0.998	0.363	0.400	1.998	76.960
LR	-	130	10/10/110	10,483	0.998	0.371	0.407	1.998	81.230
PI	-	58	0/0/58	9481	0.993	0.373	0.386	1.995	48.760
WBE	-	153	20/0/133	7893	1.000	0.251	0.319	1.913	150.530
WBA	-	63	18/8/37	9876	1.000	0.338	0.390	1.996	73.080
BMX	Guangxi	26	8/18/0	8426	1.000	0.365	0.351	1.971	26.450
DB	Guangxi	15	1/14/0	9329	0.984	0.371	0.368	1.966	16.260
DHB	Guangxi	32	3/29/0	7047	1.000	0.343	0.311	1.942	15.700
GZH	Guangxi	29	5/24/0	8348	1.000	0.366	0.348	1.962	32.020
LC	Guangxi	28	3/25/0	7237	0.984	0.328	0.315	1.898	34.930
LL	Guangdong	25	3/22/0	11,008	0.984	0.430	0.420	1.999	27.890
LT	Guangdong	28	6/22/0	7406	1.000	0.351	0.320	1.906	17.400
MH	Guangdong	24	6/18/0	7847	1.000	0.360	0.327	1.895	11.020
XE	Guangdong	34	0/34/0	8299	0.984	0.363	0.350	1.973	45.410
YDH	Guangdong	25	6/19/0	10,400	1.000	0.432	0.399	1.990	15.910

^1^ DR = Duroc, LW = Large White, LR = Landrace, PI = Pietrain, WBE = European wild boar, WBA = Asian wild boar, BMX = Bamaxiang, DB = Debao, DHB = Dahuabai, GZH = Guizhonghua, LC = Luchuan, LL = Longlin, LT = Lantang, MH = Meihua, XE = Guangdongxiaohua, YDH = Yuedonghei; ^2^ N_SNP_ = the number of single nucleotide polymorphisms (SNPs) with minor allele frequency (MAF) ≥ 0.2 in the 12,808 (SNP) subset; ^3^ P_N_ = the proportion of SNPs which displayed polymorphisms in the 12,808 SNPs selected from the SNP panel; ^4^ H_O_ = observed heterozygosity; ^5^ H_E_ = expected heterozygosity; ^6^ A_R_ = allelic richness; ^7^ Ne = effective population size of three generations ago.

**Table 2:** Summary of significant SNPs detected by *F_ST_* ^1^.

Chr	ID	SNP	Position, bp	*F_ST_*	Genes	QTL ^2^ (Counts)
1	rs80947145	ASGA0004899	149,872,523	0.852	*C1H18orf63*, *CYB5A*, *TIMM21*, *FBXO15*	Drip loss (15)
8	rs81261671	MARC0076384	54,466,796	0.845	--	Mean corpuscular volume (3)
8	rs81339817	ALGA0111390	55,931,429	0.838	*SRP72*, *ARL9*, *THEGL*, *HOPX*, *SPINK2*, *REST*	Mean corpuscular volume (4)
5	rs81383780	ALGA0031742	36,506,086	0.834	*TRHDE*	Meat color b* (4)
5	rs80983312	MARC0046863	44,931,786	0.831	--	Average backfat thickness (4)
7	rs80995372	ALGA0045445	125,434,386	0.825	--	Shoulder subcutaneous fat thickness (3)

^1^ The top six significant potential selection regions are shown here, and all significant potential selection regions are shown in Table S3. ^2^ QTL = Quantitative trait locus, the traits with the highest QTL count are shown here, and all QTLs can be seen in Table S3.

**Table 3:** Gene Ontology (GO) terms and Kyoto Encyclopedia of Genes and Genomes (KEGG) pathways enriched with candidate genes ^1.^

Term	Count	Genes	*p*-Value
ssc00592:alpha-Linolenic acid metabolism	6	*PLA2G12A*, *PLA2G12B*, *PLA2G2C*, *PLA2G2D*, *PLA2G5*, *PLA2G2F*	0.001
ssc04975:Fat digestion and absorption	7	*PLA2G12A*, *PLA2G12B*, *FABP1*, *PLA2G2C*, *PLA2G2D*, *PLA2G5*, *PLA2G2F*	0.002
GO:0050482~arachidonic acid secretion	5	*PLA2G12A*, *PLA2G12B*, *PLA2G2C*, *PLA2G2D*, *PLA2G2F*	0.002
GO:0007338~single fertilization	7	*PSP-II*, *RNASE10*, *PSP-I*, *AWN*, *AQN-1*, *SMAD4*, *SPMI*	0.003
GO:0090501~RNA phosphodiester bond hydrolysis	3	*ANG*, *RNASE4*, *RNASE6*	0.005

^1^ The top six GO terms and KEGG pathways are shown here, and all significant potential selection regions are in Table S5.

## Supplementary Materials

The following are available online at https://www.mdpi.com/2076-2615/9/6/361/s1, Figure S1: Minor allele frequency distribution of each breed, Figure S2: Genetic distance distribution of pair-wise individuals; Figure S3: Principal component analysis results of each South China indigenous individual; Figure S4: The likelihood of K in structure analysis; Figure S5: Structure results among South China indigenous pig breeds; Figure S6: The likelihood of K in structure analysis among South China indigenous pig breeds; Figure S7: The predicted $r^{2}$ of different distances and true $r^{2}$; Table S1: The genetic distance of each individual; Table S2: Parameters of linkage disequilibrium decay model of each breed; Table S3: Candidate genes and Quantitative Trait Locus (QTLs) overlapped with potential selection regions; Table S4: *F_ST_* of all SNPs; Table S5: Gene Ontology (GO) terms and Kyoto Encyclopedia of Genes and Genomes (KEGG) pathways enriched with candidate genes.
