# Supplementary figures and images for: Genetic Diversity of Indigenous Pigs from South China Area Revealed by SNP Array
Source: Animals (Basel). 2019 Jun 16;9(6):361. doi: 10.3390/ani9060361 (PMC6616596; doi:10.3390/ani9060361)

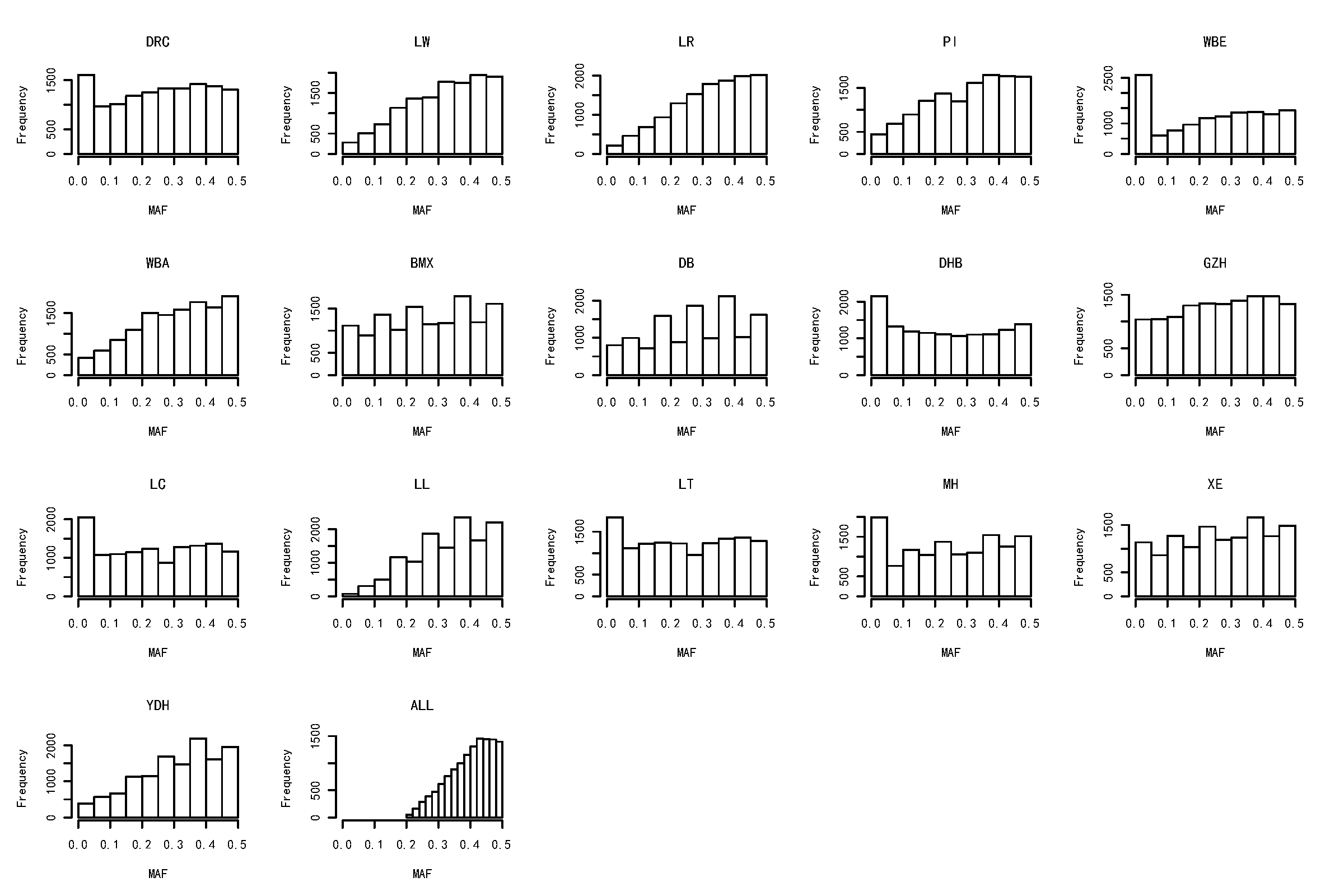

Supplement: Supplementary file 1 [file animals-09-00361-s001.zip › Supplementary Materials-animals-498952/Figure S1 Minor allele frequency distribution of each breed.png]

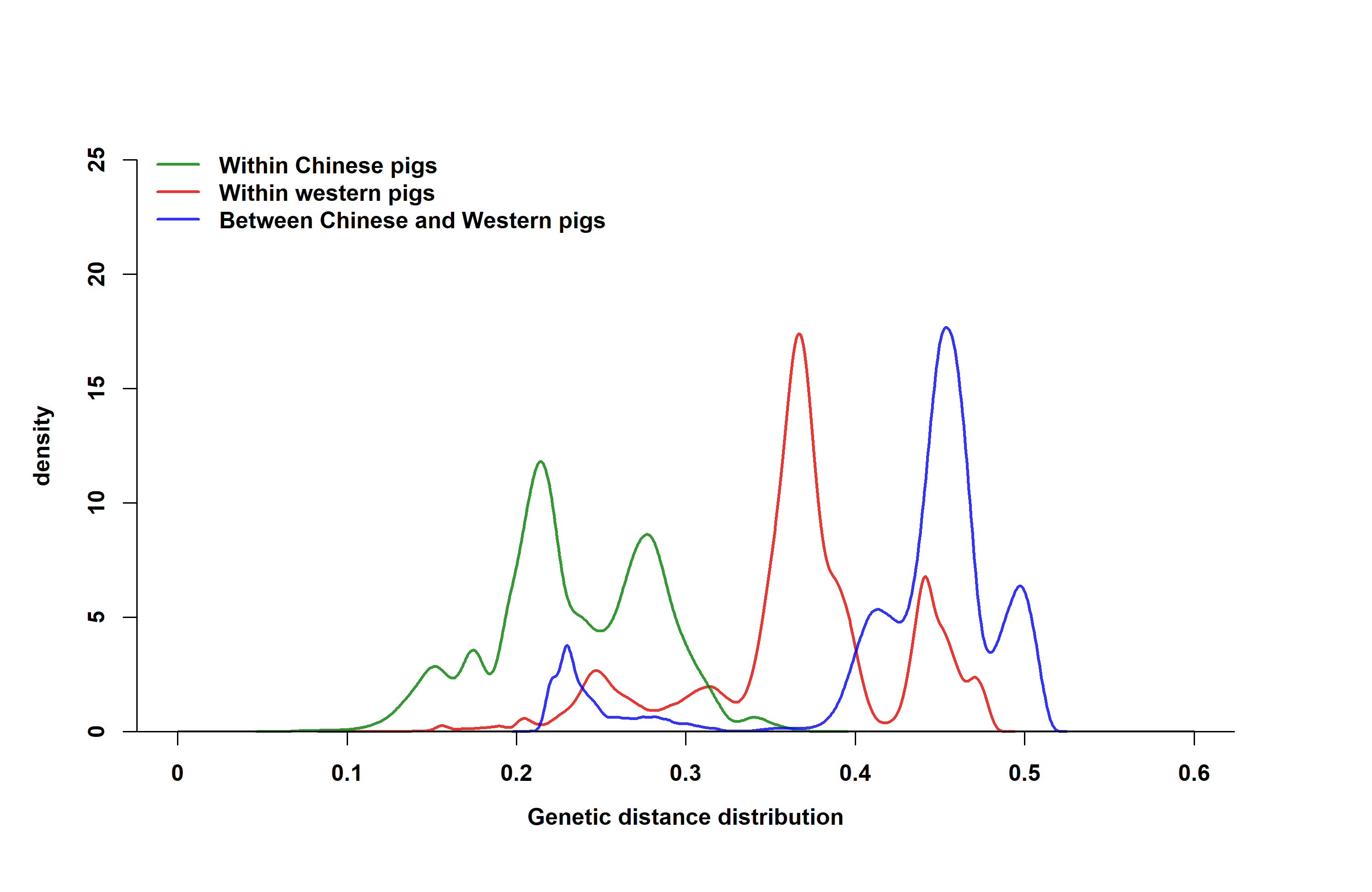

Supplement: Supplementary file 1 [file animals-09-00361-s001.zip › Supplementary Materials-animals-498952/Figure S2 Genetic distance distribution of pair-wise individuals.png]

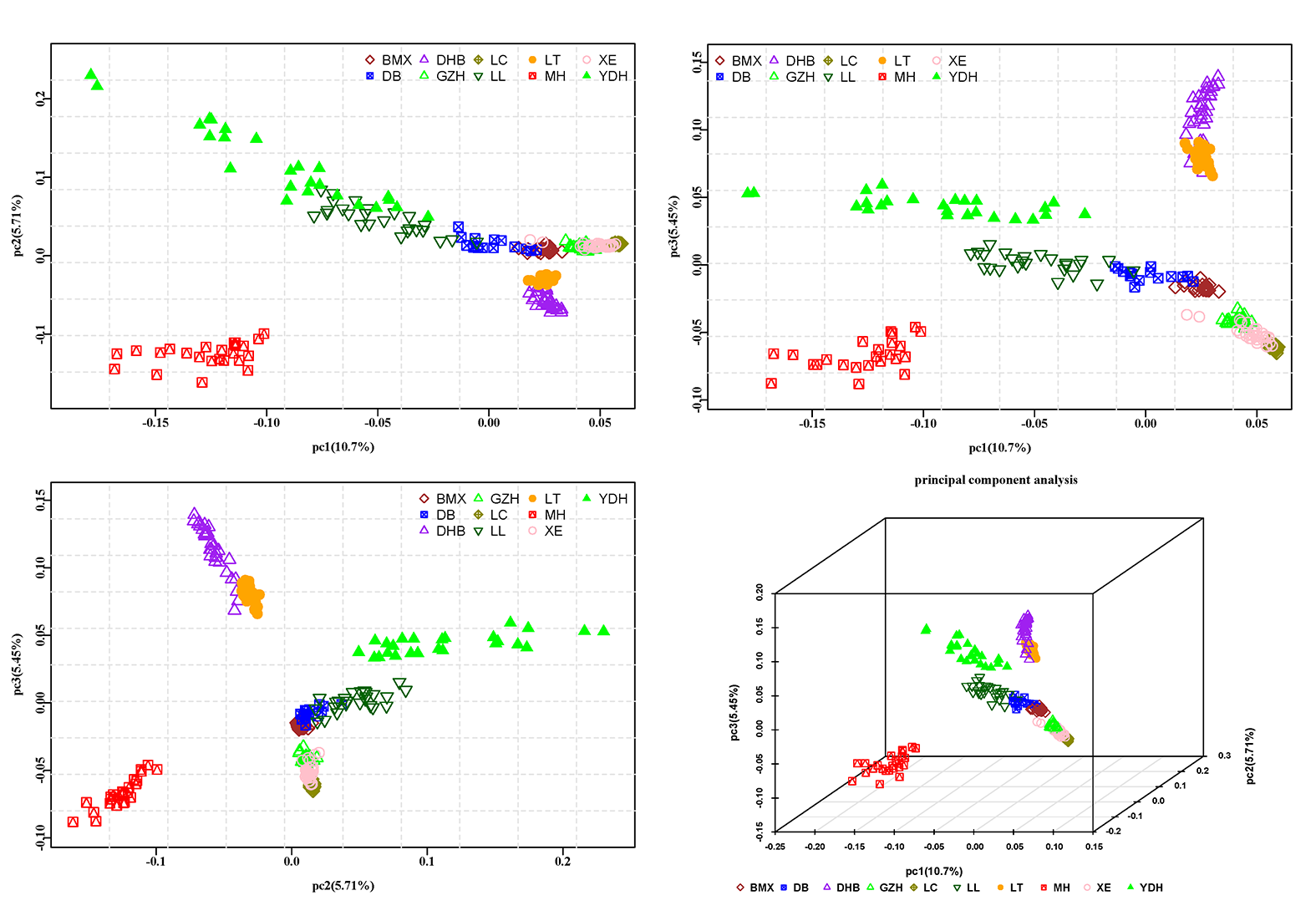

Supplement: Supplementary file 1 [file animals-09-00361-s001.zip › Supplementary Materials-animals-498952/Figure S3 Principal component analysis results of each South China indigenous individual.tif]

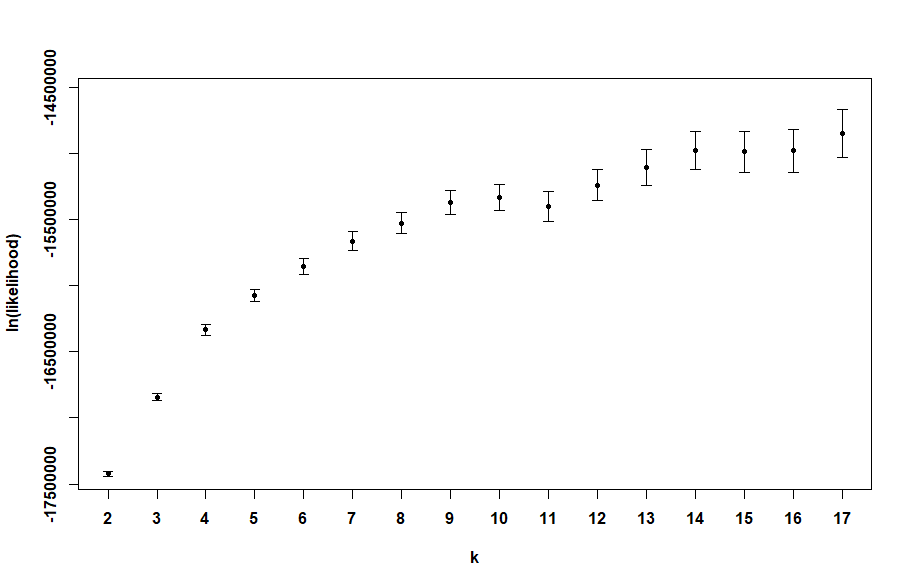

Supplement: Supplementary file 1 [file animals-09-00361-s001.zip › Supplementary Materials-animals-498952/Figure S4 The likelihood of K in structure analysis.png]

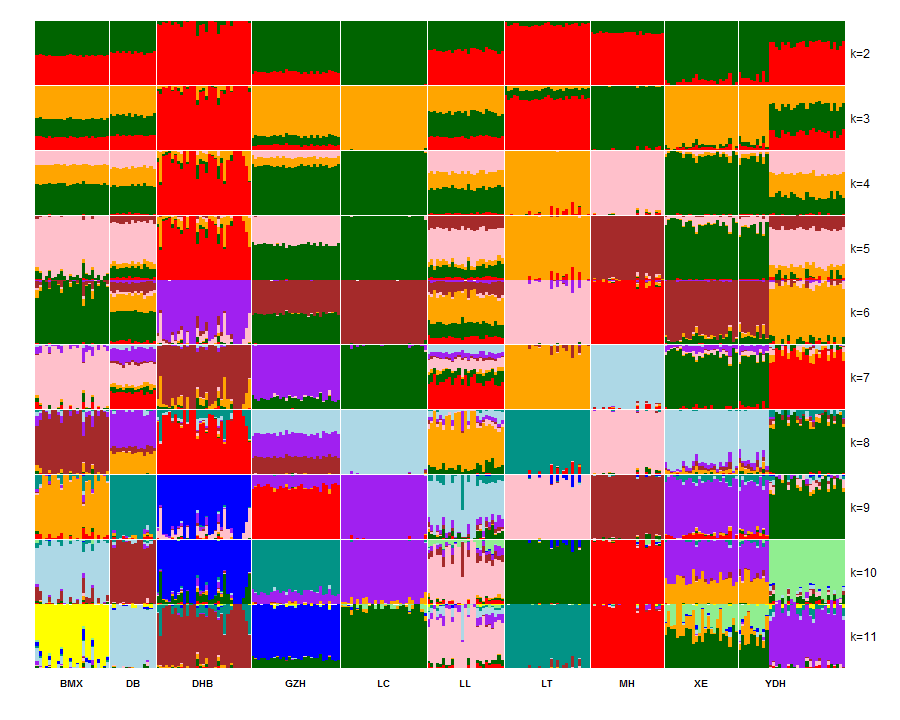

Supplement: Supplementary file 1 [file animals-09-00361-s001.zip › Supplementary Materials-animals-498952/Figure S5 Structure results among South China indigenous pig breeds.tiff]

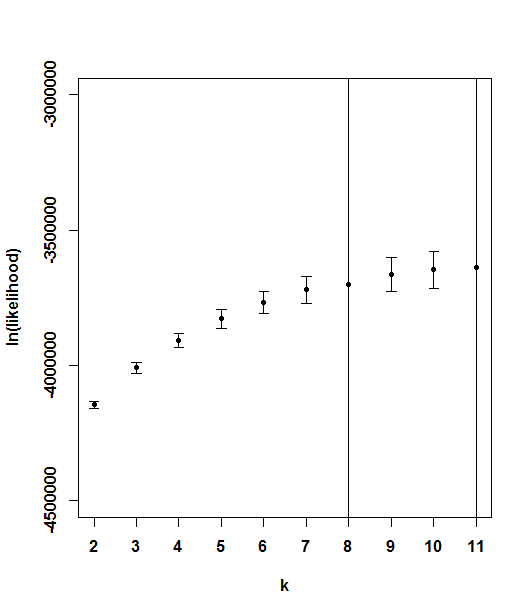

Supplement: Supplementary file 1 [file animals-09-00361-s001.zip › Supplementary Materials-animals-498952/Figure S6 The likelihood of K in structure analysis among South China indigenous pig breeds.png]

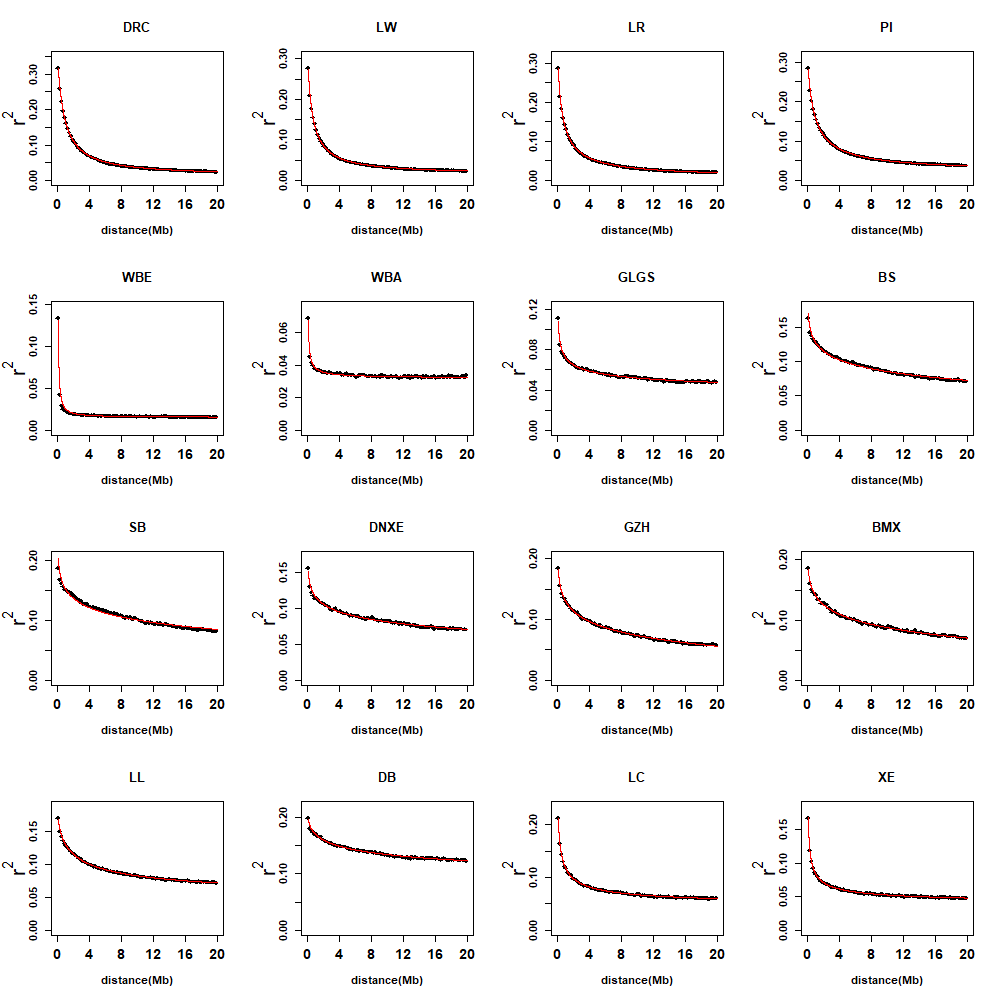

Supplement: Supplementary file 1 [file animals-09-00361-s001.zip › Supplementary Materials-animals-498952/Figure S7 The predicted r2 of different distances and true r2.png]
